# Análise de Custo-Efetividade da Terapia com Evolocumabe em Pacientes com Alto Risco de Eventos Cardiovasculares no Contexto do SUS – Brasil

**DOI:** 10.36660/abc.20200690

**Published:** 2021-11-01

**Authors:** Luiza Latado, Rodrigo Morel Vieira de Melo, Sóstenes Mistro, Adriana Lopes Latado, Harrison Floriano do Nascimento, Yasmin Menezes Lira, Natalia Ferreira Cardoso de Oliveira, Yuri de Santana Galindo, Tainá Viana, Luiz Carlos Santana Passos

**Affiliations:** 1 Universidade Federal da Bahia Faculdade de Medicina Salvador BA Brasil Universidade Federal da Bahia - Faculdade de Medicina, Salvador, BA – Brasil; 2 Hospital Ana Nery Serviço de Cardiologia Salvador BA Brasil Hospital Ana Nery - Serviço de Cardiologia, Salvador, BA – Brasil; 3 Universidade Federal da Bahia Programa de Pós-Graduação em Saúde Coletiva Instituto Multidisciplinar em Saúde Vitória da Conquista BA Brasil Universidade Federal da Bahia. Instituto Multidisciplinar em Saúde - Programa de Pós-Graduação em Saúde Coletiva, Vitória da Conquista, BA - Brasil; 4 Hospital Universitário Professor Edgard Santos Pesquisa e Inovação Tecnológica Salvador BA Brasil Hospital Universitário Professor Edgard Santos - Pesquisa e Inovação Tecnológica, Salvador, BA – Brasil; 5 Escola Bahiana de Medicina e Saúde Pública Salvador BA Brasil Escola Bahiana de Medicina e Saúde Pública, Salvador, BA – Brasil

**Keywords:** Doença da Artéria Coronariana/tratamento farmacológico, Saúde Pública, Evolocumabe, Análise Custo-Benefício/economia, Hipercolesterolemia/terapia, Medição de Risco, Infarto do Miocárdio

## Abstract

**Fundamento:**

Em associação às estatinas, os inibidores da pró-proteína convertase subtilisina/kexina tipo 9 (PCSK9) demonstraram ser eficazes na redução de eventos cardiovasculares em pacientes de alto risco.

**Objetivo:**

Analisar a custo-efetividade da implementação de evolocumabe para pacientes com alto risco de eventos cardiovasculares no contexto do Sistema Único de Saúde (SUS) no Brasil.

**Métodos:**

Um modelo de Markov foi utilizado, baseando-se em uma amostra ambulatorial de pacientes com doença arterial coronariana. Os desfechos primários analisados foram infarto agudo do miocárdio, acidente vascular cerebral isquêmico (AVCi), revascularização do miocárdio e morte cardiovascular. O resultado foi expresso por meio da razão de custo-efetividade incremental (RCEI), considerando-se uma taxa de desconto de 5% ao ano, e uma análise de sensibilidade foi realizada, tendo em vista a imprecisão de valores.

**Resultados:**

Selecionaram-se 61 pacientes com risco cardiovascular estimado em 35% em 10 anos, se em uso de atorvastatina 80mg/dia, e em 22,75%, se adicionado o evolocumabe. O custo global por paciente no período de 10 anos foi de R$ 46.522,44 no grupo em monoterapia com atorvastatina *versus* R$ 236.141,85 na terapia combinada, com uma efetividade global de 0,54 e 0,73, respectivamente. Isso resultou em uma RCEI R$ 1.011.188,07 (R$ 864.498,95 a R$ 1.296.748,43) por desfecho cardiovascular evitado.

**Conclusões:**

Apesar de não existirem padrões nacionais para custo-efetividade, os dados encontrados sugerem que a estratégia de associação do evolocumabe à terapia com estatina não é, no momento, custo-efetiva.

## Introdução

As doenças cardiovasculares (DCV) são a principal causa de mortalidade no Brasil e no mundo.^1^ No Brasil, respondem por 29% dos óbitos em indivíduos ≥ 20 anos, conforme estudo feito pelo Departamento de Informática do Sistema Único de Saúde (Datasus) em 2015.^2^ Entre as DCVs, destaca-se a aterosclerose, uma doença com patogenia intrinsicamente relacionada a fatores de risco modificáveis ou não.^3^

Altos níveis da lipoproteína de baixa densidade (LDL-c) ocupam papel de destaque no risco da doença aterosclerótica. As terapias hipocolesterolêmicas para redução do LDL-c são fundamentais nesse cenário, e as estatinas mostraram-se eficazes e efetivas na prevenção de desfechos cardiovasculares.^4^ Estima-se que, para cada 39mg/dL de diminuição de LDL-c com estatinas, ocorra uma redução relativa de eventos cardiovasculares maiores na ordem de 21%.^5^

Os inibidores da pró-proteína convertase subtilisina/kexina tipo 9 (PCSK9) são uma nova classe de medicação para hipercolesterolemia, que tem como representantes comercializados no Brasil o evolocumabe e o alirocumabe. A PCSK9 é uma protease capaz de inibir a reciclagem dos receptores de LDL-c (LDL-R) expressos na superfície dos hepatócitos, diminuindo a captação hepática de LDL-c e elevando seus níveis plasmáticos.^6^ Por consequência, a inibição da PCSK9 possibilita a reciclagem dos LDL-R e aumenta a depuração do LDL-c circulante.

O estudo FOURIER demonstrou uma redução adicional de 59% nos níveis de LDL-c e de 15% nos desfechos cardiovasculares com o uso de evolocumabe (comparado com placebo) em pacientes de risco cardiovascular elevado, já em uso de estatina.^7^ Conforme as atualizações das diretrizes de especialidades, o evolocumabe é recomendado para a prevenção secundária de eventos em pacientes tratados com estatina de alta potência e que não tenham alcançado os níveis de LDL-c preconizados.^8^

Análises econômicas acerca da utilização desses novos fármacos são ainda muito escassas; porém, extremamente necessárias uma vez que seu custo direto é muito elevado. Um recente estudo americano demonstrou que o evolocumabe não foi custo-efetivo quando comparado ao uso isolado de estatinas.^9^ O presente estudo tem como objetivo avaliar a custo-efetividade do uso do evolocumabe em relação à terapia-padrão para pacientes com alto risco de eventos cardiovasculares acompanhados no sistema de saúde público brasileiro.

## Métodos

### Delineamento e amostragem

Trata-se de um estudo de avaliação econômica do tipo custo-efetividade, que comparou atorvastatina 80mg/dia, considerada a terapia hipolipemiante padrão, com a atorvastatina 80mg/dia combinada ao evolocumabe 140mg/mL a cada 15 dias na redução estimada de eventos cardiovasculares ateroscleróticos em pacientes com história prévia de síndrome coronariana aguda (SCA). Custos e benefícios foram avaliados para a perspectiva da sociedade, sobretudo no contexto do sistema público de saúde brasileiro.

O modelo econômico do estudo foi aplicado sob uma amostra por conveniência, obtida a partir de uma coorte prospectiva de pacientes em prevenção secundária e acompanhados no ambulatório de doença arterial coronariana (DAC) em um hospital público de referência na cidade de Salvador, na Bahia. Os critérios de inclusão dessa coorte foram SCA há menos de 1 ano, associada a falha em alcançar meta de LDL menor que 50mg/dL em tratamento convencional com estatina de alta potência, com ou sem ezetimibe, pelo período mínimo de 12 semanas. Foram considerados critérios de exclusão: doença concomitante fora de perspectiva terapêutica, sobrevida estimada inferior a 1 ano e participação de outro protocolo de pesquisa semelhante. Aplicaram-se os critérios de elegibilidade apenas nos pacientes que concordaram em participar do estudo e assinaram termo de consentimento livre e esclarecido.

Dessa coorte foram selecionados para estudo os pacientes que adicionalmente preenchiam os critérios de elegibilidade do ensaio clínico FOURIER:^7^ idade entre 40 e 85 anos, LDL-c ≥70mg/dL e uso otimizado de estatina de alta potência ou, no mínimo, dose diária de 20mg de atorvastatina.

### Análise estatística

A estatística descritiva foi utilizada para resumir as variáveis de interesse da amostra. Empregou-se o teste de Kolmogorov-Smirnov para verificar a normalidade das variáveis contínuas, com valores de p > 0,05, indicando distribuição normal. As variáveis contínuas com distribuição normal foram descritas pelas médias e desvios-padrão. Descreveram-se as variáveis categóricas por seu valor absoluto e percentual.

### Modelo econômico

Os pacientes incluídos no estudo tiveram seu risco de desfechos decorrentes da doença aterosclerótica estratificado em 10 anos, de acordo com a presença de comorbidades e conforme publicação prévia.^10^ Considerou-se a categoria de maior risco em que o paciente se enquadrava, e o risco foi estimado por meio do cálculo da média do intervalo de risco, como descrito na Tabela 1.

**Tabela 1:** Categorias de alto risco para doença cardiovascular em 10 anos para pacientes em terapia com estatina, baseadas em dados de ensaios clínicos publicados

Categoria	Risco projetado em 10 anos (%)
Doença cardiovascular aterosclerótica clínica + diabetes	28-38
Com DRC	28-43
Sem DRC	26-29
Doença cardiovascular aterosclerótica clínica + DRC	34-35
SCA recente (< 3 meses)	32
DAC + fatores de risco mal controlados	28-41
DAC + Doença vascular periférica	43-55
DAC + ≥ 65 anos	21-54
AVCi/ataque isquêmico transitório e homem	31
DAC + hipercolesterolemia familiar (LDL-c ≥ 190mg/dL)	41

DRC: doença renal crônica; SCA: síndrome coronariana aguda; DAC: doença arterial coronariana; AVCi: acidente vascular cerebral isquêmico. Adaptado de Robinson et al.^10^

A partir do risco estimado em 10 anos e de uma intervenção hipotética para a redução de eventos cardiovasculares com o inibidor de PCSK9 nesses pacientes, elaborou-se um modelo de redução de risco cardiovascular com o evolocumabe para a amostra em estudo. Esse modelo baseou-se em dados do ensaio clínico FOURIER,^7^ que demonstraram uma redução adicional de 59% do LDL-c com o evolocumabe em pacientes já em uso de estatinas e em dados da metanálise CTT^5^ (*Cholesterol treatment trialists*). Constatou-se que, para cada 39 mg/dL de diminuição do valor de LDL-c, ocorreu uma redução no número de eventos cardiovasculares maiores de 21%. Apesar de o estudo FOURIER apresentar um seguimento de 26 meses, os resultados encontrados foram extrapolados para o período de 10 anos no presente estudo.

**Figura 1:**
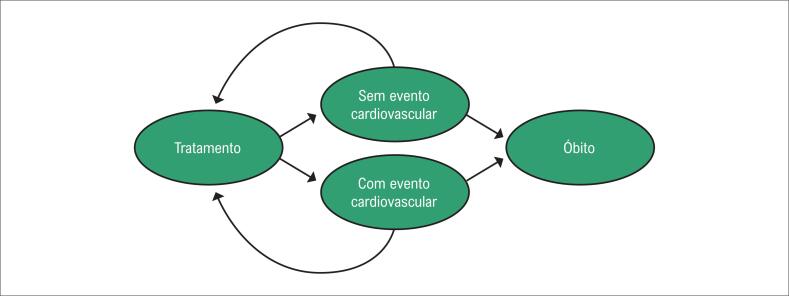
Representação esquemática do modelo de Markov utilizado na comparação entre atorvastatina 80mg versus atorvastatina + evolocumabe. CV: cardiovascular.

A avaliação de custo-efetividade foi realizada por meio de um modelo de Markov representado na Figura 1, que utilizou como desfecho primário uma combinação de eventos cardiovasculares maiores: infarto agudo do miocárdio (IAM); acidente vascular cerebral isquêmico (AVCi); revascularização do miocárdio (RM); e morte cardiovascular. Apesar de Robinson et al.,^10^ não considerarem a RM como um dos desfechos avaliados, entende-se que intervenções coronarianas são frequentemente realizadas no pós-IAM, e uma vez que seu custo não está incluso no pagamento da internação por IAM, tal desfecho foi considerado para a análise.^11^

**Figura 2:**
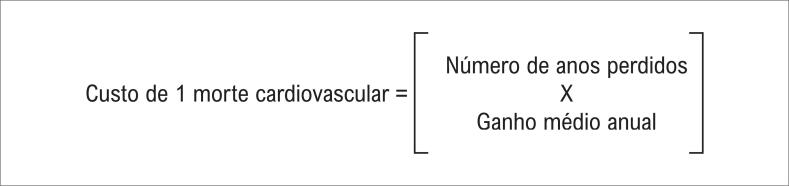
Fórmula utilizada para estimativa de custo da morte cardiovascular. Adaptado de Siqueira et al.^11^

Os custos de internamento por IAM, AVCi e RM foram obtidos através do Sistema de Gerenciamento da Tabela de Procedimentos, Medicamentos e OPM (Sigtap) do SUS, e coletaram-se os custos diretos relativos aos medicamentos a partir de dados da Secretaria de Saúde do Estado da Bahia.^12^ Os custos indiretos referentes à morte cardiovascular precoce foram calculados de acordo com o esquema demonstrado na Figura 2. O cálculo foi efetuado por meio da multiplicação do número de anos perdidos devido à morte precoce, considerando a expectativa de vida média do brasileiro e a média de idade da população avaliada pelo ganho financeiro anual médio do brasileiro. O salário utilizado no presente estudo foi a média salarial da população brasileira em 2017, corrigida para a taxa de desemprego no mesmo período. Tais dados foram obtidos por meio do Instituto Brasileiro de Geografia e Estatística (IBGE).^13^

Estimaram-se os custos referentes ao tratamento com a estatina de alta potência a partir do valor de aquisição, via atacado pela nossa instituição, de uma unidade de comprimido de atorvastatina na dosagem de 40mg. No que diz respeito ao evolocumabe, pelo fato de não ser uma medicação adquirida no contexto do SUS, utilizou-se o valor de comercialização no varejo de uma seringa unitária na dosagem de 140mg.

Os resultados foram apresentados por meio da razão custo-efetividade incremental (RCEI), definida como o custo adicional da terapia com evolocumabe, expresso em reais (R$), dividido pelo ganho adicional em saúde alcançado, expresso por desfecho cardiovascular evitado, quando comparado com a terapia-padrão com atorvastatina de alta potência. Para o cálculo, considerou-se uma taxa de desconto de 5% ao ano.

### Análise de sensibilidade

Para avaliar a robustez do modelo, realizaram-se análises de sensibilidade determinística e probabilística. Na análise determinística, os parâmetros do modelo variaram em até 20% para mais ou para menos, a fim de obter uma faixa RCEI. A análise probabilística foi realizada para avaliar a incerteza dos valores RCEI calculados. Para tanto, conduziu-se uma análise de Monte Carlo por microssimulação com 1.000 tentativas aleatórias. A partir dessa análise, gerou-se a curva de aceitabilidade para avaliar a probabilidade de que um tratamento seja mais custo-efetivo que outro, como função-limite da disposição a pagar por uma unidade adicional de efetividade. As análises foram realizadas no software *TreeAge Pro 2020 R.2*.

### Considerações éticas

Conforme a Resolução 466/2012 do Conselho Nacional de Saúde, o presente estudo foi aprovado pelo Comitê de Ética em pesquisa local, CAAE nº 68053317.9.0000.0045. Todos os procedimentos foram realizados de acordo com a declaração de Helsinki.

## Resultados

De acordo com os critérios de inclusão, avaliaram-se 61 pacientes no presente estudo, e suas características clínicas e demográficas foram comparadas às da população acompanhada pelo estudo FOURIER, demonstrando uma heterogeneidade moderada entre os dois grupos, conforme descrito na Tabela 2. A amostra apresentava média de idade de 63 (±11) anos, 32 indivíduos (52%) eram do sexo masculino e tinha como fator de risco cardiovascular mais prevalente a hipertensão arterial sistêmica (83%), seguido do *diabetes mellitus* (42%) e do tabagismo (31%). Desses pacientes, 54% sofreram IAM prévio e apresentavam uma média de LDL-c de 111(±34) mg/dL, dos quais 57% apresentavam valor de LDL-c maior ou igual a 100 mg/dL.

**Tabela 2:** Características clínicas e demográficas da população de pacientes com doença arterial coronariana e no ensaio FOURIER

	AMOSTRA	FOURIER
Idade, média (±DP)	63 (11)	63 (9)
Sexo masculino, n° (%)	32 (52)	20.795 (75)
**Fatores de risco cardiovascular, n° (%)**		
Hipertensão	51 (83)	22.040 (80)
Diabetes melito	26 (42)	9.333 (34)
Tabagismo	19 (31)	7.770 (28)
**História de doença vascular, n° (%)**		
IAM	33 (54)	22.356 (71)
AVCi	0 (0)	5.330 (17)
Uso de ezetimibe, n° (%)	6 (10)	1.393 (5)
**Parâmetros lipídicos**		
LDL-c, média (±DP), mg/dL	111 (34)	97 (28)
LDL-c 70-99 mg/dL, n° (%)	26 (43)	15.586 (57)
LDL-c ≥ 100mg/dL, n° (%)	35 (57)	9.943 (36)
HDL-c, média (±DP), mg/dL	45 (13)	46 (13)
Triglicerídeos, média (±DP), mg/dL	159 (97)	149 (70)

DAC: doença arterial coronariana; AVCi: acidente vascular cerebral isquêmico.

O risco individual médio de IAM, AVCi, RM ou morte cardiovascular em 10 anos dos pacientes do estudo, quando em uso isolado de terapia com atorvastatina, foi de 35%. Os custos de internamento por IAM, AVC isquêmico e RM foram, respectivamente, R$ 588,12, R$ 463,21 e R$ 6.756,37, enquanto o valor de um comprimido de atorvastatina 40mg foi de R$ 1,00 e o de uma seringa de 140 mg de evolocumabe foi de R$ 901,61.

Para o cálculo do custo da morte cardiovascular precoce, consideraram-se a média de idade de 63 anos dos pacientes e a de morte de 68 anos, considerando que, em um período de 10 anos, o óbito ocorreria, em média, após 5 anos. Adequando-se à proporção de homens e mulheres, a expectativa média de vida da amostra estudada foi de 75 anos e 8 meses, levando a uma perda de 7,7 anos de vida caso o evento “morte” ocorresse, e o ganho médio anual corrigido para taxa de desemprego foi de R$ 22.128,00. Dessa maneira, uma morte cardiovascular precoce na população estudada teria o custo de R$ 170.385,60.

Na estimativa realizada, o tratamento com evolocumabe reduziria a média de LDL-c da população de 111 mg/dL para 45,5 mg/dL, o que representaria uma redução relativa de risco de 35% em relação ao uso isolado de atorvastatina 80 mg/dia. Assim, os pacientes em uso da terapia combinada atorvastatina e evolocumabe teriam um risco individual de 22,75% de ocorrência de um dos eventos que compõem o desfecho composto (IAM, AVCi, RM ou morte cardiovascular em 10 anos), representando uma redução de risco absoluto projetada em 10 anos de 12,25%. No cálculo da média dos custos referentes a cada um dos desfechos, observando-se a proporção de ocorrência dos mesmos no grupo placebo do estudo FOURIER,^7^ obteve-se um valor médio de R$ 23.145,40, caso um dos desfechos ocorresse.

O custo com o medicamento referente à terapia-padrão com atorvastatina 80mg/dia por 10 anos seria de R$ 7.300,00 por paciente tratado, enquanto na terapia com atorvastatina 80mg/dia + evolocumabe 140mg administrado a cada 15 dias seria de R$ 223.686,40 por paciente durante 10 anos. Ao considerar o custo global por paciente, que inclui a probabilidade de ocorrência e os custos dos desfechos negativos, o custo global do tratamento com atorvastatina em monoterapia foi de R$ 46.522,44 *versus* R$ 236.141,85 na terapia combinada, com uma efetividade global de 0,54 e 0,73, respectivamente.

**Figura 3:**
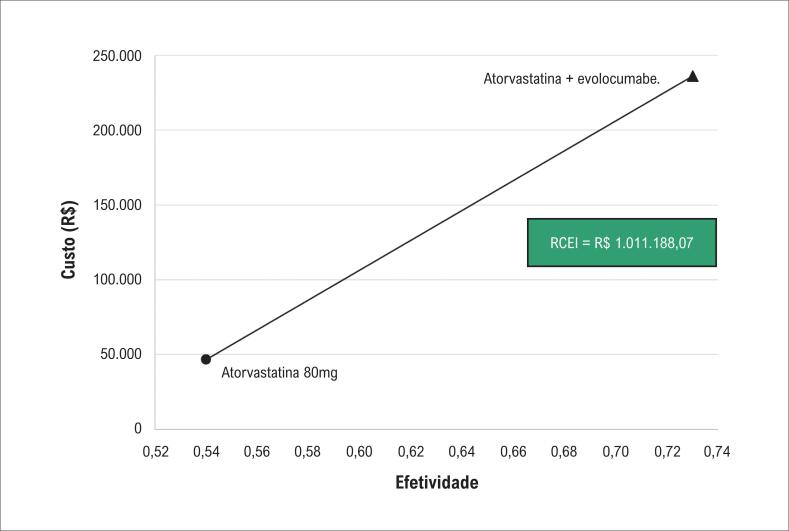
Comparação de custo-efetividade entre atorvastatina e atorvastatina + evolocumabe na redução de desfechos cardiovasculares. RCEI: razão de custo-efetividade incremental.

Ao considerar a média dos custos e efetividades observadas no modelo, obtiveram-se um custo incremental de R$ 189.619,41 e uma efetividade incremental de 0,19, o que resultou em uma RCEI de R$ 1.011.188,07 por desfecho cardiovascular evitado. A Figura 3 resume a comparação da relação custo-efetividade entre as duas alternativas analisadas no estudo.

Na Tabela 3, é possível observar os resultados das medidas de custo e de efetividade resultantes do modelo econômico, com a respectiva análise de sensibilidade obtida através da simulação de Monte Carlo.

**Tabela 3:** Simulação de Monte Carlo na avaliação de custo-efetividade da terapia combinada de atorvastatina e evolocumabe versus terapia-padrão com atorvastatina em monoterapia

		Tratamento	
Atributo	Medida	Atorvastatina	Atorvastatina + evolocumabe
**Custo (R$)**			
	Média	46.122,35	220.373,82
	Desvio-padrão	2.136,05	1.450,45
	Mediana	46.065,31	220.404,32
	Percentil 2,5	41.643,23	217.668,81
	Percentil 10	43.402,22	218.484,95
	Percentil 90	48.845,06	222.212,71
	Percentil 97,5	50.186,16	223.240,95
**Efetividade**			
	Média	0,55	0,73
	Desvio-padrão	0,01	0,01
	Mediana	0,55	0,73
	Percentil 2,5	0,53	0,72
	Percentil 10	0,54	0,72
	Percentil 90	0,56	0,74
	Percentil 97,5	0,56	0,75

**Figura 4:**
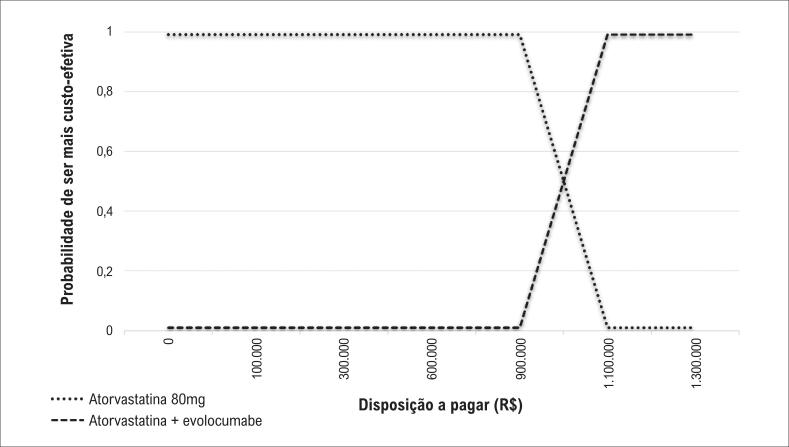
Curva de aceitabilidade em função da disposição a pagar na comparação entre atorvastatina versus atorvastatina + evolocumabe na redução de desfechos cardiovasculares.

Na análise de sensibilidade determinística, com variação dos valores de custo e de efetividade de cada uma das estratégias, obteve-se uma faixa de variação da RCEI de R$ 864.498,95 a R$ 1.296.748,43. Pela análise da curva de aceitabilidade (Figura 4), foi possível observar que, apenas após um incremento de R$ 1.000.000,00 na disponibilidade a pagar, a terapia associada com evolocumabe apresentou maior probabilidade de ser mais custo-efetiva.

## Discussão

No presente estudo, um modelo de redução de risco cardiovascular demonstrado pelo ensaio clínico FOURIER foi extrapolado para o período de 10 anos e utilizado para avaliar a custo-efetividade da adição do evolocumabe em amostra acompanhada no SUS. Os pacientes eram portadores DAC comprovada, com SCA recente e níveis elevados de LDL-c, apesar de estarem em terapia otimizada com estatina de alta potência. A análise de custo-efetividade demonstrou que a adição do evolocumabe 140mg a cada 15 dias à terapia-padrão, considerando o atual valor de compra de ambos medicamentos, acarretaria um custo incremental em 10 anos de R$ 189.619,41 por paciente. Dessa forma, seria necessário investir R$ 1.011.188,07 com a terapia adicional do evolocumabe para cada evento cardiovascular (fatal ou não) adicional evitado.

Os inibidores de PCSK9 surgiram como uma terapia promissora para pacientes com alto risco de eventos cardiovasculares, em prevenção secundária e níveis elevados de LDL-c refratários à terapia com estatina de alta potência, apresentando uma redução absoluta de risco maior e um número necessário para tratar (NNT) menor em pacientes com níveis mais altos de LDL-c residual.^14^ No entanto, cada vez mais, compreende-se a importância de uma análise econômica em saúde prévia à tomada de decisão acerca da implementação de novas tecnologias, inclusive de medicamentos, no sistema público de saúde, uma vez que novas tecnologias quase sempre estão acompanhadas de incrementos financeiros elevados ao sistema. Esse conhecimento permitiria que a alocação de recursos econômicos fosse realizada de uma forma mais sistemática do que intuitiva pelos gestores da saúde.^15^ Assim, em relação ao evolocumabe, um anticorpo monoclonal humanizado, estudos deste tipo são necessários para tomada de decisão sobre sua implementação no SUS.

Muitos países, na tentativa de padronizar um valor para orientar as decisões de incorporação de novas tecnologias aos sistemas de saúde, têm estabelecido um limiar de custo-efetividade, este representado por uma razão entre custo monetário no numerador e medida de ganho em saúde no denominador, valor que pode variar, e abaixo do qual uma tecnologia é considerada custo-efetiva. No Brasil, o Ministério da Saúde ainda não estabeleceu um limiar de custo-efetividade.^16^ A utilização de valores estabelecidos por outros países em estudos nacionais é questionável, visto que a definição do limiar é contexto-específica, dependendo da riqueza local, disponibilidade e capacidade de pagar, características do sistema de saúde e das preferências sociais.^17^ Estudos publicados no Brasil, no entanto, utilizaram-se do limiar de custo-efetividade sugerido pela Organização Mundial da Saúde (OMS) de três vezes o Produto Interno Bruto (PIB) *per capita* por anos de vida ajustados para qualidade de vida (QALY), mesmo sem utilização da mesma medida de ganho em saúde.^18^ Desse modo, caso comparássemos o resultado do presente estudo com o limiar sugerido pela OMS (R$ 95.500,50/QALY, considerando o PIB *per capita* do Brasil em 2017), teríamos um resultado sem custo-efetivo.

Apesar disso, existem experiências similares na literatura. Um estudo realizado nos Estados Unidos (2017) com o objetivo de avaliar a custo-efetividade do evolocumabe em pacientes com doença aterosclerótica cardíaca concluiu que a adição do inibidor de PCSK9 à terapia-padrão hipolipemiante acarretaria um custo incremental de US$ 105.398,00 e ganho em QALY de 0,39, o que representaria uma RCEI de US$ 268.637,00 por QALY ganho, ultrapassando o limiar de US$ 150.000,00 por QALY utilizado pelo estudo.^9^ Apesar de a unidade de ganho em saúde considerada pela presente análise ter sido distinta, por tratar-se de estudos com características populacionais e metodológicas similares, caso tivesse sido considerada QALY como medida de ganho em saúde, acredita-se que o evolocumabe não seria custo-efetivo no SUS, ultrapassando o limiar de US$ 150.000,00.

Na Espanha, por outro lado, um estudo realizado em 2017 avaliou a custo-efetividade do evolocumabe em dois subgrupos: pacientes com hipercolesterolemia familiar (HF) e pacientes em prevenção secundária para eventos cardiovasculares. Considerou-se um limiar de € 30.000,00 a € 45.000,00 por QALY ganho. Os resultados do estudo demonstraram uma RCEI de € 30.893,00 para o grupo HF e de € 45.340,00 para o grupo em prevenção secundária, concluindo que a adição do evolocumabe à terapia-padrão com estatina pode ser considerada uma alternativa custo-efetiva para esses subgrupos no contexto do Sistema Nacional de Saúde Espanhol.^19^ O resultado favorável à implementação do evolocumabe é, provavelmente, explicado pelos altos valores atribuídos às internações decorrentes dos desfechos cardiovasculares. Comparado à tabela utilizada pelo SUS para reembolso de internações, o valor considerado pelo estudo espanhol foi de 47 vezes o valor tabelado para IAM, 110 vezes o valor para AVCi e 8 vezes o valor para RM.

Uma metanálise publicada em 2019 avaliou a custo-efetividade dos inibidores de PCSK9 na doença cardiovascular, analisando 16 estudos realizados em diferentes países, com resultados estimados para toda a vida.^20^ O estudo encontrou uma grande variação nos limiares de custo-efetividade considerados e nos custos anuais da terapia com inibidores de PCSK9. Os valores de RCEI variavam de US$ 51.687,00 a US$1.336.221,00, no que se detectou a necessidade de redução de 20% a 88% nos valores de mercado dos inibidores de PCSK9 para que a terapia seja considerada custo-efetiva. Dessa forma, assim como sugerido no presente estudo, apesar de sua eficácia comprovada, o alto custo da terapia com os inibidores de PCSK9 a torna não custo-efetiva na população, de modo geral. Reduções no preço do fármaco foram implementadas em alguns países. Faz-se necessário que novas análises sejam realizadas, considerando a redução do custo com a terapia.

No contexto nacional, é importante ressaltar o subfinanciamento crônico do Sistema Único de Saúde, que pode, pelo menos em parte, justificar os resultados encontrados. Um exemplo claro é a relação de valores subestimados e encontrados na Tabela SUS, o padrão de referência para o pagamento dos serviços prestados por estabelecimentos que atendem a rede pública de saúde. Esses valores, preestabelecidos, muitas vezes não cobrem os reais custos pela prestação de um serviço ou realização de um procedimento,^21^ o que pode ser parcialmente explicado pela defasagem que não acompanhou os índices inflacionários dos últimos anos. Dessa forma, o impacto financeiro da redução das internações por IAM, AVCi e RM pela adição do evolocumabe poderia ser maior. Consequentemente, isso acarretaria um custo incremental menor, pois o alto gasto com a adição do evolocumabe à terapia-padrão seria contrapesado por uma maior economia financeira, por conta da prevenção dos desfechos cardiovasculares.

Neste sentido, deve-se levar em consideração que os custos com o tratamento-padrão com atorvastatina foram estimados a partir do seu valor no atacado, através da aquisição na nossa instituição, que é financiada pelo sistema único de saúde. Por outro lado, os custos relacionados ao evolocumabe foram obtidos a partir do seu valor de comercialização no varejo. Levando isso em consideração, acreditamos que variações dos valores de custos estão contempladas na análise de sensibilidade realizada, apresentando uma margem inferior de RCEI de R$ 864.498,95, ainda muito elevado para demonstrar custo-efetividade da terapia.

O estudo apresenta outras limitações. Inicialmente, enquanto o estudo FOURIER avaliou a prevenção de desfechos cardiovasculares em um seguimento médio de 26 meses, os valores encontrados foram extrapolados para um período de 10 anos. Durante esse período, caso os benefícios na prevenção dos desfechos diferissem do estudo FOURIER ou efeitos adversos significantes ocorressem, a estimativa de custo-efetividade poderia ser alterada. De fato, observou-se uma diminuição progressiva nos eventos cardiovasculares ao longo do ensaio clínico; portanto, o benefício total do evolocumabe na redução de eventos cardiovasculares pode ter sido subestimado.

Uma potencial limitação, no sentido de não ter sido considerado o valor referente à antecipação da aposentadoria no cálculo do custo dos desfechos avaliados, não é aplicável, uma vez que a média de idade da amostra de pacientes é superior à idade média de aposentadoria por tempo de contribuição (55,6 anos para homens e 52,8 anos para mulheres). Segundo dados do INSS de 2018, não há impacto financeiro no caso de evolução com incapacidade laborativa ou óbito precoce, além dos estimados pela redução do PIB. A ausência de um limiar de custo-efetividade brasileiro bem estabelecido e com unidade de ganho em saúde similar à utilizada no presente estudo dificultou que fosse concluído com exatidão se a estratégia é ou não custo-efetiva. Além disso, a análise econômica do evolocumabe baseou-se em uma amostra específica de pacientes em prevenção secundária e de alto risco para eventos cardiovasculares, não devendo ser extrapolada para o cenário de prevenção primária ou outras populações de menor risco cardiovascular.

## Conclusão

Apesar de não existirem padrões nacionais para aceitabilidade nas análises de custo-efetividade, os dados encontrados sugerem que a estratégia de associação do evolocumabe à terapia com estatina não é, no momento, custo-efetiva. A redução dos valores do tratamento e/ou a seleção de candidatos à terapia com maior perfil de risco ajudariam a alcançar melhores valores de custo-efetividade. Diante disso, futuras discussões sobre o tema devem envolver profissionais de saúde e gestores do SUS, avaliando-se grupos de pacientes com maior risco cardiovascular, de modo a tornar possível a disponibilização de terapias eficazes para melhorar a saúde da população.
