# Errata

**DOI:** 10.36660/abc.20240582

**Published:** 2024-10-18

**Edição de Novembro de 2021, vol. 117(5), págs. 988-996**

No Artigo Original “Análise de Custo-Efetividade da Terapia com Evolocumabe em Pacientes com Alto Risco de Eventos Cardiovasculares no Contexto do SUS – Brasil”, com número de DOI: https: https://doi.org/10.36660/abc.20200690, publicado no periódico Arquivos Brasileiros de Cardiologia, Arq Bras Cardiol. 2021; 117(5):988-996, na página 1, alterar o nome da autora Luiza Latado para: Luisa Latado.

