# Supplementary material for: Análise de Custo-Efetividade da Terapia com Evolocumabe em Pacientes com Alto Risco de Eventos Cardiovasculares no Contexto do SUS – Brasil
Source: Arq Bras Cardiol. 2021 Nov 1;117(5):988–96. [Article in Portuguese] doi: 10.36660/abc.20200690 (PMC8682111; doi:10.36660/abc.20200690)
Supplement: Translation

# Translation: Cost-Effectiveness Analysis of Evolocumab Therapy in Patients at High Risk of Cardiovascular Events in the Context of the Brazilian Unified Health System

**Authors:** Luiza Latado, Rodrigo Morel Vieira de Melo, Sóstenes Mistro, Adriana Lopes Latado, Harrison Floriano do Nascimento, Yasmin Menezes Lira, Natalia Ferreira Cardoso de Oliveira, Yuri de Santana Galindo, Tainá Viana, Luiz Carlos Santana Passos

**Affiliations:** 1 Salvador BA Brazil Universidade Federal da Bahia - Faculdade de Medicina, Salvador, BA – Brazil; 2 Salvador BA Brazil Hospital Ana Nery - Serviço de Cardiologia, Salvador, BA – Brazil; 3 Vitória da Conquista BA Brazil Universidade Federal da Bahia. Instituto Multidisciplinar em Saúde - Programa de Pós-Graduação em Saúde Coletiva, Vitória da Conquista, BA - Brazil; 4 Salvador BA Brazil Hospital Universitário Professor Edgard Santos - Pesquisa e Inovação Tecnológica, Salvador, BA – Brazil; 5 Salvador BA Brazil Escola Bahiana de Medicina e Saúde Pública, Salvador, BA – Brazil

**Keywords:** Cardiomyopathy, Hypertrophic, Hypertension, Echocardiography/methods, Magnetic Resonance Spectroscopy/methods, Left Ventricular Hypertrophy

## Abstract

**Background:**

Hypertrophic cardiomyopathy (HCM) and left ventricular hypertrophy (LVH) secondary to systemic hypertension (HTN) may be associated with left atrial (LA) functional abnormalities.

**Objectives:**

We aimed to characterize LA mechanics in HCM and HTN and determine any correlation with the extent of left ventricular (LV) fibrosis measured by cardiac magnetic resonance (CMR) in HCM patients.

**Methods:**

Two-dimensional speckle tracking-derived longitudinal LA function was acquired from apical views in 60 HCM patients, 60 HTN patients, and 34 age-matched controls. HCM patients also underwent CMR, with measurement of late gadolinium enhancement (LGE) extension. Association with LA strain parameters was analyzed. Statistical significance was set at p<0.05.

**Results:**

Mean LV ejection fraction was not different between the groups. The E/e’ ratio was impaired in the HCM group and preserved in the control group. LA mechanics was significantly reduced in HCM, compared to the HTN group. LA strain rate in reservoir (LASRr) and in contractile (LASRct) phases were the best discriminators of HCM, with an area under the curve (AUC) of 0.8, followed by LA strain in reservoir phase (LASr) (AUC 0.76). LASRr and LASR-ct had high specificity (89% and 91%, respectively) and LASr had sensitivity of 80%. A decrease in 2.79% of LA strain rate in conduit phase (LASRcd) predicted an increase of 1cm in LGE extension (r^2^=0.42, β 2.79, p=0.027).

**Conclusions:**

LASRr and LASRct were the best discriminators for LVH secondary to HCM. LASRcd predicted the degree of LV fibrosis assessed by CMR. These findings suggest that LA mechanics is a potential predictor of disease severity in HCM.

## Introduction

Cardiovascular diseases (CVDs) are the main cause of mortality in Brazil and in the world.^1^ In Brazil, they account for 29% of deaths in individuals ≥ 20 years old, according to the Informatics Department of the Brazilian Unified Health System (DATASUS), in 2015.^2^ Among the CVDs, atherosclerotic cardiovascular disease (ASCVD) stands out: a disease of which pathogeny is intrinsically related to modifiable or non-modifiable risk factors.^3^

High levels of low-density lipoprotein (LDL) cholesterol play an important role in the ASCVD risk. Lipid-lowering therapies to reduce LDL levels are essential in this scenario, and statins are efficient and effective in preventing cardiovascular outcomes.^4^ It is estimated that, for each 39mg / dL decrease in LDL cholesterol with statins, there is a relative reduction in major cardiovascular events in the order of 21%.^5^

Pro-protein convertase subtilisin-kexin type 9 (PCSK9) inhibitors are a new class of medication for hypercholesterolemia, represented in the Brazilian market by evolocumab and alirocumab. PCSK9 is a protease capable of inhibiting the recycling of LDL receptors (LDL-R) expressed on the surface of hepatocytes, decreasing the hepatic uptake of LDL and increasing its plasma levels.^6^ Consequently, the inhibition of PCSK9 enables the recycling of LDL-R and increases the clearance of circulating LDL-cholesterol.

The FOURIER study demonstrated an additional 59% reduction in LDL cholesterol levels and 15% in cardiovascular outcomes with the use of evolocumab (compared with placebo) in patients at high cardiovascular risk already using statins.^7^ According to updates of the specialty guidelines, evolocumab is recommended for the secondary prevention of events in patients treated with a high-potency statin who have not reached the recommended LDL cholesterol levels.^8^

Economic analyses of the use of these new drugs are still very scarce but extremely necessary, since their direct cost is very high. A recent North-American study showed that evolocumab was not cost-effective when compared to statin use alone.^9^ The present study aims to evaluate the cost-effectiveness of using evolocumab in comparison to the standard therapy for patients at high risk of cardiovascular events monitored in the Brazilian Unified Health System (SUS, *Sistema Único de Saúde*).

## Methods

### Study design and sampling

This is a cost-effectiveness economic evaluation study that compared the standard lipid-lowering therapy with atorvastatin 80 mg/day *versus* atorvastatin 80 mg/day combined with evolocumab 140 mg/mL every 15 days, in the estimated reduction of cardiovascular atherosclerotic events in patients with a previous history of acute coronary syndrome (ACS). Costs and benefits were assessed from the perspective of society, particularly in the context of the Brazilian public health system.

The economic model of the study was applied using a convenience sample obtained from a prospective cohort of patients undergoing secondary prevention followed at the coronary artery disease (CAD) outpatient clinic in a public referral hospital in the state capital city of Salvador, state of Bahia, Brazil. The inclusion criteria for this cohort were ACS occurring less than 1 year ago, associated with failure to achieve an LDL target of less than 50 mg/dL under conventional treatment with a high-potency statin, with or without ezetimibe, for at least 12 weeks. The exclusion criteria included: concomitant disease outside the therapeutic perspective, estimated survival of less than 1 year, and participation in another similar research protocol. The eligibility criteria were applied only to patients who agreed to participate in the study and signed an informed free and informed consent form.

From this cohort, patients who additionally met the eligibility criteria for the FOURIER^7^ clinical trial were selected for the study, namely: age between 40 and 85 years, LDL-cholesterol level ≥ 70 mg/dL, and optimized use of a high-potency statin or, at least, 20mg daily dose of atorvastatin.

### Statistical analysis

Descriptive statistics were used to summarize the variables of interest in the sample. The Kolmogorov-Smirnov test was used to verify the normality of continuous variables, with p values > 0.05 indicating a normal distribution. Continuous variables with normal distribution were described as means and standard deviations, and categorical variables were described as their absolute and percentage values.

### Economic model

The patients included in the study had their risk of outcomes resulting from ASCVD stratified in 10 years, according to the presence of comorbidities, as shown in a previous publication.^10^ The highest risk category in which the patient was classified was considered, and the risk was estimated by calculating the average of the risk interval, as described in Table 1.

**Table 1:** High-risk categories for cardiovascular disease at 10 years for patients on statin therapy, based on published clinical trial data

Category	Projected risk over 10 years (%)
Clinical ASCVD + diabetes	28-38
With chronic kidney disease	28-43
Without chronic kidney disease	26-29
Clinical ASCVD + chronic kidney disease	34-35
Recent ACS (<3 months)	32
CAD + poorly controlled risk factors	28-41
CAD + Peripheral vascular disease	43-55
CAD + ≥ 65 years old	21-54
IS/Transient ischemic attack	31
CAD + Familial hypercholesterolemia (LDL cholesterol ≥ 190mg/dL)	41

ASCVD: atherosclerotic cardiovascular disease; ACS: acute coronary syndrome; CAD: coronary artery disease; IS: ischemic stroke. Adapted from Robinson et al.^10^

Based on the estimated 10-year risk and a hypothetical intervention to reduce cardiovascular events with the PCSK9 inhibitor in these patients, a cardiovascular risk reduction model was developed with evolocumab for the study sample. This model was based on data from the FOURIER^7^ clinical trial, which demonstrated an additional 59% reduction in LDL levels with evolocumab in patients already using statins, and data from the CTT^5^ (Cholesterol Treatment Trialists) meta-analysis, which found that for every 39 mg/dL of decrease in the LDL-cholesterol value, there was a reduction in the number of cardiovascular events greater than 21%. Although the FOURIER study has a 26-month follow-up, the observed results were extrapolated to the 10-year period in the present study.

The cost-effectiveness assessment was performed using a Markov model, as depicted in Figure 1, which used as a primary outcome the combination of major cardiovascular events: non-fatal myocardial infarction (MI); non-fatal ischemic stroke (IS), coronary revascularization (RV); and cardiovascular death. Although Robinson et al.^10^ do not consider RV as one of the evaluated outcomes, it is understood that coronary interventions are often performed after a MI, and, since its cost is not included in the payment for hospitalization for AM, this outcome was considered for the analysis.

**Figure 1:** Schematic representation of the Markov Model used in the comparison between Atorvastatin 80mg versus Atorvastatin + Evolocumab.

The hospitalization costs for MI, IS and RV were obtained through the Management System of the List of Procedures, Medication, and Orthotics/Prosthetics and Special Materials - OPM (SIGTAP) of SUS, while the direct costs related to medication were obtained from data from the State Health Department of the state of Bahia.^11^ The indirect costs related to early cardiovascular death were calculated according to the schematic representation shown in figure 2. The calculation was made by multiplying the number of years lost due to early death, considering the average life expectancy of the Brazilian individual and the average age of the assessed population, by the average annual financial gain of the Brazilian individual.^12^ The salary used in this study was the average wage of the Brazilian population in 2017 corrected for the unemployment rate in the same period. Data was obtained through the Brazilian Institute of Geography and Statistics (IBGE, *Instituto Brasileiro de Geografia e Estatística*).^13^

**Figure 2:** Formula used to estimate the cost of cardiovascular death. Adapted from Siqueira et al.^11^

The costs related to the treatment with high-potency statins were estimated based on the wholesale purchase price by our institution of a unit of atorvastatin tablet in the dose of 40mg. Regarding evolocumab, since it is not a medication acquired in the context of SUS, the retail price of a syringe unit at the dose of 140mg was used.

The results were presented using the Incremental Cost-Effectiveness Ratio (ICER), defined as the additional cost of the therapy with evolocumab, expressed in R$, divided by the additional achieved health gain, expressed by avoided cardiovascular outcome, when compared with standard therapy with high potency atorvastatin. For the calculation, a discount rate of 5% per year was considered.

### Sensitivity analysis

To assess the robustness of the model, deterministic and probabilistic sensitivity analyses were performed. In the deterministic analysis, the parameters of the model were varied by up to 20% more or less, to obtain a range of ICER variation. The probabilistic analysis was performed to assess the uncertainty of the ICER calculated values. To this end, a Monte Carlo analysis was conducted by microsimulation including 1,000 random attempts. From this analysis, the acceptability curve was generated to assess the probability that one treatment is more cost-effective than another, as a limit function of the willingness to pay for an additional unit of effectiveness. The analyses were performed using the TreeAge Pro 2020 R.2 software.

### Ethical considerations

According to resolution 466/2012 of the National Health Council, the present study was approved by the local research ethics committee, CAAE number 68053317.9.0000.0045, and all procedures were performed in accordance with the declaration of Helsinki.

## Results

According to the inclusion criteria, 61 patients were evaluated in the present study and their clinical and demographic characteristics were compared to those of the population monitored by the FOURIER study, demonstrating a moderate heterogeneity between the two groups, as shown in table 2. The sample had a mean age of 63 (±11) years old, 32 (52%) were males and the most prevalent cardiovascular risk factors were hypertension (83%), followed by diabetes mellitus (42%) and smoking (31%). Of these patients, 54% had suffered a previous MI and had an average LDL-cholesterol level of 111 (±34) mg/dL, with 57% of them having an LDL value ≥100 mg/dL.

**Table 2:** Clinical and demographic characteristics of the population of patients with coronary artery disease and in the FOURIER trial

	SAMPLE	FOURIER
Age, mean (±SD)	63 (11)	63 (9)
Male, N. (%)	32 (52)	20,795 (75)
**Cardiovascular risk factors, N. (%)**
Hypertension	51 (83)	22,040 (80)
Diabetes Mellitus	26 (42)	9,333 (34)
Smoking	19 (31)	7,770 (28)
**Previous vascular disease, N. (%)**
MI	33 (54)	22,356 (71)
IS	0 (0)	5,330 (17)
Ezetimibe use, N. (%)	6 (10)	1,393 (5)
**Lipid parameters**
LDL cholesterol, mean (±SD), mg/dL	111 (34)	97 (28)
LDL cholesterol 70-99 mg/dL, No. (%)	26 (43)	15,586 (57)
LDL cholesterol ≥ 100mg/dL, No. (%)	35 (57)	9,943 (36)
HDL cholesterol, mean (±SD), mg/dL	45 (13)	46 (13)
Triglycerides, mean (±SD), mg/dL	159 (97)	149 (70)

SD: standard deviation; MI: myocardial infarction; IS: ischemic stroke.

The average individual 10-year risk of MI, IS, RV or cardiovascular death among the study patients was 35%, if using isolated therapy with atorvastatin. The costs of hospitalization for MI, IS and RV were, respectively, R$ 588.12, R$ 463.21, and R$ 6,756.37, while the value of an atorvastatin 40mg tablet was R$ 1.00 and that of a 140 mg syringe of evolocumab was R$ 901.61.

To calculate the cost of early cardiovascular death, the mean age of patients was 63 years old and the average age of death was 68 years considering that, in a period of 10 years, death would occur, on average, after 5 years. Adjusting to the proportion of men and women, the average life expectancy of the study sample was 75 years and 8 months with a loss of 7.7 years of life if the death event occurred, and the average annual gain corrected for the unemployment rate was R$ 22,128.00. Thus, an early cardiovascular death in the studied population would cost R$ 170,385.60.

According to the estimate, treatment with evolocumab would reduce the average LDL-cholesterol level of the population from 111 mg/dL to 45.5 mg/dL, which would represent a relative risk reduction of 35% in comparison to the isolated use of atorvastatin 80mg/day. Thus, patients using the combined therapy of atorvastatin and evolocumab would have an individual risk of 22.75% of the occurrence of one of the events that constitute the composite outcome (MI, IS, RV, or cardiovascular death in 10 years), representing an absolute risk reduction projected over 10 years of 12.25%. When calculating the average costs for each of the outcomes, observing the proportion of their occurrence in the placebo group of the FOURIER^7^ study, an average value of R$ 23,145.40 was obtained, if one of the outcomes occurred.

The cost of the drug for standard therapy with atorvastatin 80mg/day for 10 years would be R$ 7,300.00 per treated patient, while it would be R$ 223,686.40 per patient for 10 years for therapy with atorvastatin 80mg/day + evolocumab 140mg administered every 15 days. When considering the global cost per patient, which includes the probability of occurrence and the costs of negative outcomes, the global cost of treatment with atorvastatin monotherapy was R$ 46,522.44, *versus* R$ 236,141.85 for the combined therapy, with an overall effectiveness of 0.54 and 0.73, respectively.

When considering the average costs and effectiveness observed in the model, an incremental cost of R$ 189,619.41 and incremental effectiveness of 0.19 were obtained, which resulted in an ICER of R$ 1,011,188.07 for an avoided cardiovascular outcome. Figure 3 summarizes the comparison of the cost-effectiveness ratio between the two alternatives analyzed in the study.

**Figure 3:** Cost-effectiveness comparison between Atorvastatin and Atorvastatin + Evolocumab in reducing cardiovascular outcomes. ICER: Incremental Cost-Effectiveness Ratio.

Table 3 shows the results of the cost and effectiveness measures resulting from the economic model, with the respective sensitivity analysis obtained through the Monte Carlo simulation.

**Table 3:** Monte Carlo simulation in the cost-effectiveness assessment of the combined therapy of atorvastatin and evolocumab versus standard therapy with atorvastatin alone

		Therapy	
Attribute	Measure	Atorvastatin	Atorvastatin + Evolocumab
**Cost (R$)**			
	Mean	46,122.35	220,373.82
	Standard deviation	2,136.05	1,450.45
	Median	46,065.31	220,404.32
	2.5^th^ Percentile	41,643.23	217,668.81
	10^th^ Percentile	43,402.22	218,484.95
	90^th^ Percentile	48,845.06	222.212.71
	97.5^th^Percentile	50,186.16	223,240.95
**Effectiveness**			
	Mean	0.55	0.73
	Standard deviation	0.01	0.01
	Median	0.55	0.73
	2.5^th^ Percentile	0.53	0.72
	10^th^ Percentile	0.54	0.72
	90^th^ Percentile	0.56	0.74
	97.5^th^ Percentile	0.56	0.75

In the deterministic sensitivity analysis, with variation in the cost and effectiveness values of each of the strategies, a range of ICER variation was obtained, from R$ 864,498.95 to R$ 1,296,748.43 and through the analysis of the acceptability (Figure 4), it was possible to observe that the combined therapy with evolocumab was more likely to be more cost-effective only after an increase of R$ 1,000,000.00 in the availability to pay.

**Figure 4:** Acceptability curve according to the willingness to pay when comparing Atorvastatin versus Atorvastatin + Evolocumab in reducing cardiovascular outcomes.

## Discussion

In the present study, a cardiovascular risk reduction model demonstrated by the FOURIER clinical trial was extrapolated to 10 years and used to assess the cost-effectiveness of adding evolocumab to a sample monitored through SUS. The patients had proven CAD, with recent ACS and elevated LDL-cholesterol levels, despite optimized high-potency statin therapy. The cost-effectiveness analysis showed that adding evolocumab 140mg every 15 days to the standard therapy, considering the current purchase value of both drugs, would result in an incremental cost in 10 years of R$ 189,619.41 per patient. Thus, it would be necessary to invest R$ 1,011,188.07 with additional evolocumab therapy for each additional cardiovascular event (fatal or not) avoided in the sample.

PCSK9 inhibitors have emerged as a promising therapy in the secondary prevention for patients at high risk of cardiovascular events, and with high levels of LDL- cholesterol refractory to high-potency statin therapy, with a greater absolute risk reduction and a lower number needed treat (NNT) in patients with higher residual levels of LDL-cholesterol.^14^ However, the importance of the economic analysis in health before deciding about the implementation of new technologies, including medications, in the public health system is increasingly understood, since new technologies are almost always accompanied by high financial increments to the system. This knowledge would allow the allocation of economic resources to be carried out in a more systematic than intuitive way by health managers.^15^ Thus, concerning evolocumab, a humanized monoclonal antibody, studies like this are necessary to decide about its implementation in SUS.

Many countries, aiming to standardize a value to guide decisions about the incorporation of new technologies into health systems, have established a cost-effectiveness threshold. This is represented by a ratio, between the monetary cost in the numerator and the measure of health gain in the denominator, a measure that can vary, below which the technology is considered cost-effective. In Brazil, the Ministry of Health has not yet established a cost-effectiveness threshold.^16^ The use of values established by other countries in national studies is questionable, since the definition of the threshold is context-specific depending on the local wealth, availability and ability to pay, characteristics of the health system, and social preferences.^17^ Studies published in Brazil, however, have already used the cost-effectiveness threshold suggested by the World Health Organization (WHO) of three times the Gross Domestic Product (GDP) *per capita* for years of life using the quality-adjusted life year (QALY), even if not using the same measure of health gain.^18^ So, if we compared the result of this study with the threshold suggested by the WHO (R$ 95,500.00/QALY, considering Brazil's GDP *per capita* in 2017), we would have a non-cost-effective result.

Despite this, there are similar experiences in the literature. A study carried out in the United States (2017) intending to evaluate the cost-effectiveness of evolocumab in patients with ASCVD concluded that adding PCSK9 inhibitor to the standard lipid-lowering therapy would result in an incremental cost of U$ 105,398.00 and an increase in QALY of 0.39. This would represent an ICER of U$ 268,637.00 per achieved QALY, which exceeds the threshold of U$ 150,000.00 per QALY used by the study.^9^ Even though the health gain unit considered by the present analysis was distinct, since it deals with studies with similar population and methodological characteristics, if QALY were considered the measure of health gain, it is believed that evolocumab would not be cost-effective in SUS, as it exceeds the threshold of U$ 150,000.00.

In Spain, on the other hand, a study carried out in 2017 evaluated the cost-effectiveness of evolocumab in two subgroups: patients with familial hypercholesterolemia (FH) and patients undergoing secondary prevention for cardiovascular events. A threshold of € 30,000.00 to € 45,000.00 per achieved QALY was considered. The results of the study demonstrated an ICER of € 30,893.00 for the HF group and € 45,340.00 for the secondary prevention group, concluding that the addition of evolocumab to the standard statin therapy can be considered a cost-effective alternative for these subgroups in the context of the Spanish National Health System.^19^ This favorable result for the implementation of evolocumab is probably explained by the high values attributed to hospitalizations resulting from cardiovascular outcomes. Compared with the list used by SUS for hospitalization reimbursement, the value considered by the Spanish study was 47 times the tabulated value for MI, 110 times the value for IS, and 8 times the value for RV.

A meta-analysis published in 2019 assessed the cost-effectiveness of PCSK9 inhibitors in cardiovascular disease, analyzing 16 studies carried out in different countries with estimated results for life.^20^ The study found a wide variation in the considered cost-effectiveness thresholds and in the annual costs of therapy with PCSK9 inhibitors, with ICER values ranging from U$ 51,687 to U$ 1,336,221 and the need for a 20% to 88% reduction in the purchase values of PCSK9 inhibitors for the therapy to be considered cost-effective. Thus, as suggested in the present study, despite its proven efficacy, the high cost of therapy with PCSK9 inhibitors makes it non-cost-effective for the general population. Reductions in the price of the drug have been implemented in some countries and it is necessary for further analysis to be carried out, considering the decrease in the cost of therapy.

In the national context, it is important to highlight the chronic underfunding of SUS, which can, at least in part, justify the observed results. A clear example is the underestimated values found in the SUS list: the reference standard for payment for services provided by establishments that provide service to the public health network. These pre-established values often do not cover the real costs of providing a service or carrying out a procedure,^21^ which can be partly explained by the lag in the values in the SUS list that have not kept up with inflation rates in recent years. Therefore, the financial impact of reducing hospital admissions for MI, IS, and RV through the addition of evolocumab could be greater. Consequently, this would result in a lower incremental cost, since the high expense of adding evolocumab to the standard therapy would be offset by greater financial savings due to the prevention of cardiovascular outcomes.

Similarly, it should be considered that the costs of standard treatment with atorvastatin were estimated from their wholesale value, through the acquisition in our institution, which is financed by SUS. On the other hand, the costs related to evolocumab were obtained from its retail sales value. Taking this into account, we believe that variations in cost values are included in the performed sensitivity analysis, showing a lower ICER margin of R$ 864,498.95, which is still too high to demonstrate the cost-effectiveness of the therapy.

The study has other limitations. Initially, while the FOURIER study evaluated the prevention of cardiovascular outcomes during an average follow-up of 26 months, the values found were extrapolated here for a period of 10 years. During this period, if the benefits in preventing outcomes differed from the FOURIER study or if significant adverse effects occurred, the cost-effectiveness estimate could change. A progressive decrease in cardiovascular events was observed throughout the clinical trial, so the total benefit of evolocumab in reducing cardiovascular events may have been underestimated.

A potential limitation, since the amount related to early retirement was not considered in the calculation of the cost of the assessed outcomes, is not applicable. This happens, because the average age of the patient sample is older than the average retirement age by contribution time (55.6 years for men and 52.8 years for women), according to data from the Brazilian National Social Security Institute (INSS – *Instituto Nacional de Seguridade Social*) as of 2018. Thus, there is no financial impact in the case of evolution with incapacity for work or early death, in addition to those estimated by the reduction in GDP. The absence of a well-established Brazilian cost-effectiveness threshold with a health gain unit, like the one used in the present study, made it difficult to accurately conclude whether the strategy is cost-effective or not. Also, the economic analysis of evolocumab was based on a specific sample of patients undergoing secondary prevention and at high risk for cardiovascular events and should not be extrapolated to the primary prevention scenario or other populations at lower cardiovascular risk.

## Conclusion

Although there are no national standards for acceptability in cost-effectiveness analyses, the observed data suggest that the strategy of associating evolocumab with statin therapy is not cost-effective at the moment. The reduction of treatment values and/or the selection of candidates for therapy with a higher risk profile would help to achieve better cost-effectiveness values. Therefore, future discussions on the topic should involve health professionals and SUS managers assessing groups of patients at higher cardiovascular risk, allowing the availability of effective therapies to improve the population's health.
