# Supplementary material for: Errata
Source: Arq Bras Cardiol. 2024 Oct 18;121(9):e20240582. [Article in Portuguese] doi: 10.36660/abc.20240582 (PMC12092057; doi:10.36660/abc.20240582)
Supplement: Translation

# Translation: Erratum

**DOI:** 10.36660/abc.20240582i

**November 2021 Issue, vol. 117(5), pages 988-996**

In the Original Article “Cost-Effectiveness Analysis of Evolocumab Therapy in Patients at High Risk of Cardiovascular Events in the Context of the Brazilian Unified Health System”, with DOI: https://doi.org/10.36660/abc.20200690, published in the journal Arquivos Brasileiros de Cardiologia, Arq Bras Cardiol. 2021; 117(5):988-996, on page 1, change the name of the author Luiza Latado to: Luisa Latado.
